# Comparison anterior minimally invasive oblique retroperitoneal approach and posterior transpedicular approach for debridement fusion in patients with lumbar vertebral osteomyelitis: A randomized controlled trial protocol

**DOI:** 10.1097/MD.0000000000022990

**Published:** 2020-10-30

**Authors:** Xiang Gao, Shu Wan, Jie Lv, Wei Cheng, Yangbin Zhang

**Affiliations:** a Department of Orthopedics; b Department of Stomatology, The People's Hospital of Dayi County, Chengdu, Sichuan, China.

**Keywords:** oblique retroperitoneal approach, posterior transpedicular approach, protocol, vertebral osteomyelitis

## Abstract

**Background:**

Pyogenic osteomyelitis of the spine usually occurs in patients over 55 years old with acute osteomyelitis. Surgical treatment and fixation can relieve pain, enhance spinal balance and nerve function, so that patients can walk as soon as possible. Different outcomes of surgical methods include anterior minimally invasive oblique retroperitoneal approach (ORA) and posterior transpedicular approach (PTA). While, there is no consensus on the best treatment for PVO. The goal of the protocol is to compare the clinical consequences between PTA and ORA for treating PVO.

**Method:**

The experiment is a single-center randomized clinical research. This experiment was admitted by the Ethics Committee of the People's Hospital of Dayi County (Approval number: 1002-084). In all, 50 patients with lumbar vertebral osteomyelitis (LVO) who prepares surgical treatment will be included in the study. We contain adult patients (aged over 18 years) who accept debridement and spinal stabilization with LVO. Cases are removed if there is previous hardware placement, cases who are not confirmed by microbiology, or severe renal and liver dysfunction. The primary outcomes are intraoperative blood loss, operative time, hospital stay, primary failure and recurrence, and bone fusion. The secondary outcomes are postoperative pain score and physical recovery. SPSS Sample Power version 3.0 (IBM, Armonk, NY, USA) is used for data analysis.

**Results:**

Table 1 will show the outcomes in both groups.

**Conclusion:**

This protocol may offer a reliable basis for the effectiveness of the two approaches in the treatment of PVO.

**Trial registration number:**

researchregistry6046

## 1 Introduction

Pyogenic vertebral osteomyelitis (PVO) is common in acute osteomyelitis over 55 years old.^[1]^ The incidence rate of PVO in high income countries has increased gradually, increasing from 4 to 10 people in each 100 thousand population.^[2,3]^ Patients with PVO often present with nervous system damage, persistent infection and distortion.^[4,5]^ If the patient is diagnosed with early PVO, conservative treatment can obtain good results.^[6,7]^ However, patients with progressive physical instability pain, epidural abscesses, neurological damage, and antibiotic failure often need surgical operation. More and more surgeons realize that surgical treatment and fixation can reduce pain, improve spinal balance and neurologic, and lead to initial ambulation.

This paper introduces the different results of anterior minimally invasive retroperitoneal oblique approach (ORA) and posterior transpedicular approach (PTA) debridement. The ORA has the following advantages, such as less impact on nervous system, and relatively consistent access to the lower lumbar levels.^[8]^ However, in some cases involving high riding pelvis, access to the lower lumbar level is limited. Therefore, in order to perform lumbar interbody fusion from L1 to S1, the surgeon needs to separate the incision or change the position. PTA surgery is gradually accepted by surgeons. Some surgeons claimed that single-stage debridement and instrumented interbody fusion can eliminate local diseases, reduce nerve pressure, and rectify spinal malformation in single-stage surgery with smaller trauma, fewer complications, lower cost and shorter rehabilitation time.^[9,10]^ While the best treatment of PVO is still controversial, and there are few reports about it. The aim of the protocol is to compare the clinical outcomes between PTA and ORA for treating PVO.

## 2 Materials and methods

The experiment will be implemented from December 2020 to December 2021 at the People's Hospital of Dayi County, in accordance with the purposes of the Declaration of Helsinki. The experiment was admitted by the Research Ethics Committee of the People's Hospital of Dayi County (Approval number: 1002-084) and recorded in research registry (Identifier: researchregistry6046). The experiment is a single-center randomized clinical research. The recruited patients provide written informed consent prior to enrollment.

### 2.1 Inclusion and exclusion criteria

In all, 50 patients with lumbar vertebral osteomyelitis (LVO) who prepares surgical treatment will be included in the study. We include adult patients (aged above 18 years) who accept debridement and spinal stabilization with LVO. LVO has three characteristics: symptoms (back pain, fever and reduced lumbar range of motion), X-ray (vertebral and intervertebral disc lesions), and bacterial pathogens (confirmed by laboratory tests). All patients have intact medical reports. Cases are removed if there is previous hardware placement, cases who are not confirmed by microbiology, or severe renal and liver dysfunction. All patients are followed up for at least 12 months.

### 2.2 Surgical approach

(1) Procedures of ORA: patients are set in lateral decubitus position (lesion characteristics determine right or left lateral decubitus position). The retroperitoneal space is entered through a small incision of four cm. After meticulous observation with a channel and microscope system, segmental vessels are carefully examined and ligated. Then, the edge of the lesion is inspected and debridement is constructed by curette, bone biting forceps and high-speed drill. We stop debridement until normal bleeding bone appears. For patients with epidural involvement, the focus is washing with a special infusion tube. After complete resection of the lesion, titanium or peek cages are implanted to fill the bone graft/substitute.
(2) Procedures of PTA: the posterior midline straight incision is used, and the opposite side is treated with Wiltse paravertebral muscle splitting method. Place a rod to temporarily link with the screw. The lamina, facet, pedicle and proximal transverse process are excised. After meticulous observation, the vertebral body and intervertebral disc are excised by high-speed pedicle drill or bone knife to expose the normal bone endplate. After limited resection, titanium or peek cages are implanted to fill the bone graft/substitute. All patients are treated with percutaneous pedicle screw fixation.

### 2.3 Outcomes

The primary outcomes are intraoperative blood loss, operative time, hospital stay, primary failure and recurrence, and bone fusion. The secondary outcomes are postoperative pain score and physical recovery. Patient Reported Outcomes Measurement Information System^[11]^ are used to evaluate physical function.

### 2.4 Statistical analysis

SPSS Sample Power version 3.0 (IBM, Armonk, NY) is applied to analyze data. Afterward, all the data are expressed with appropriate characteristics such as mean, median, standard deviation as well as percentage. Continuous and categorical variables are analyzed using χ^2^-tests and independent t-tests, respectively. Intention to treat analysis is applied to evaluate the outcomes. Significant level: *P* < .05.

## 3 Results

Table 1 will show the outcomes in both groups.

**Table 1:**
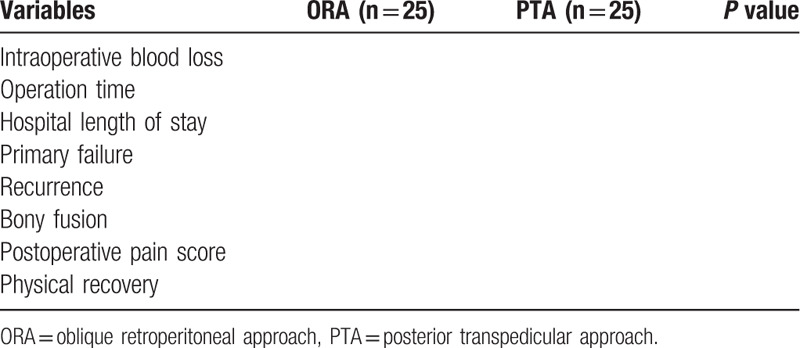
Comparison of clinical outcomes after operation.

## 4 Discussion

Many PVO patients can only use a certain amount of antibiotics. With the rapid development of neurological complications, surgical treatment is needed.^[12]^ With the continuous progress of minimally invasive method, oblique lumbar interbody fusion and lateral lumbar interbody fusion have become the option of spine surgeons.^[13,14]^ Minimally invasive ORA can directly enter the infected lesions, which is convenient for debridement.^[15,16]^ The debridement time is shorter that the amount of bleeding during operation is easy to control. In addition, the trauma to patients can be controlled through channels and microscope systems. However, thorough debridement is the central technique for the treatment of PVO. Percutaneous pedicle screws are effective for ventral decompression,^[17]^ but there are some difficulties in posterior decompression, and it is laborious to eliminate abscesses in the contralateral psoas major. Moreover, PTA can enter the infection indirectly, but it may be better than ORA in posterior decompression. Currently, no reliable evidence regarding the optimal approaches has been reported in PVO. Further investigation with large sample sizes is required.

## 5 Conclusion

This protocol may offer a reliable basis for the effectiveness of the two approaches in the treatment of PVO.

## Author contributions

**Data curation:** Shu Wan

**Funding acquisition:** Yangbin Zhang

**Investigation:** Wei Cheng

**Resources:** Jie Lv

**Software:** Jie Lv

**Writing – original draft:** Xiang Gao

**Writing – review & editing:** Wei Cheng
